# Understanding the drivers of food security among agriculture-based households in Gurué District, Central Mozambique

**DOI:** 10.1186/s40066-021-00344-3

**Published:** 2022-02-02

**Authors:** Custodio Matavel, Harry Hoffmann, Constance Rybak, Jonathan Steinke, Stefan Sieber, Klaus Müller

**Affiliations:** 1 Leibniz Centre for Agricultural Landscape Research (ZALF), Eberswalder Str. 84, 15374 Müncheberg, Germany; 2 Department of Agricultural Economics, Faculty of Life Sciences, Humboldt University of Berlin, Berlin, Germany

**Keywords:** Food consumption, Cash crops, Crop diversity, Staple food, Adequate food provisioning

## Abstract

**Background:**

The prevalence of food insecurity in Mozambique is alarming, despite progress made during the 2010s. Several studies apply different proxy indicators of food security (FS) to assess the FS situation. However, these studies overlook the factors affecting FS, using only a single data point that results in an incomplete picture of FS. Food security is expected to fluctuate, being better and worse than what studies suggest. Using a sample of 296 households to assess FS, key drivers conditioning households’ capacity to achieve FS in Gurué District, Central Mozambique, are identified. Data were collected in the pre-harvest period and during the harvest period to capture relevant interseasonal variation of FS. Household FS is assessed using three standard indicators: Household Dietary Diversity Score (HDDS), Household Food Consumption Score (HFCS), and Months of Adequate Household Food Provisioning (MAHFP).

**Results:**

Each household was classified into a specific FS status depending on the indicator applied. Generally, most households were classified as being severely or moderately food insecure during the pre-harvest season, while during the harvest season, medium and high levels of FS predominated. Nevertheless, varying outcomes were found depending on the indicator used to assess FS. MAHFP and HDDS are more related to the consumption of farm-sourced food, while HFCS responds more strongly to purchased food. Gender and age of the household head, geographic location, size and quality of land, staples production (especially cassava), livestock and crop diversity, as well as cash crops had a statistically significant effect on FS indicators.

**Conclusions:**

The study concludes that the decision whether farmers should rely on staple foods production for increasing their FS status or specialize on cash crops production to generate income and buy food depends on the indicator used to assess FS, since each indicator captures a specific domain of food security. Thus, one central recommendation derived from our results is that policy makers should promote a balance between market-oriented agriculture and subsistence production to achieve FS.

**Supplementary Information:**

The online version contains supplementary material available at 10.1186/s40066-021-00344-3.

## Background

Nearly 19% of the population in Sub-Saharan Africa (SSA) is undernourished [1]. In Mozambique, despite the progress made in reducing chronic food insecurity (FI), it is estimated at 24% [2] and 42.3% of children under the age of 5 are stunted [3]. A study conducted by [4] finds that 42–67% of households in Central Mozambique have experienced hunger during the pre-harvest period. The prevalence of undernourishment for children under the age of 5 in Gurué district, which is also located in Central Mozambique, is estimated at approximately 50% [5, 6]. Reasons for this include frequently occurring natural disasters [7], climate change [8], and destructive crop pests that hindered adequate agricultural production [9]. The latter negatively affects livelihoods in multiple ways, since approximately 80% of the economically active population work in the agricultural sector [10] and, therefore, may be at risk of FI. This situation is likely to worsen in the context of the COVID-19 crisis [11], potentially triggering irreversible long-term consequences [12].

Studies on the impacts of FI on people’s well-being indicate that it is associated with reduced physical health [13], maternal and child underweight [14], poor mental health [15], stress [16], as well as high-risk sexual behavior (e.g. in SSA) [17]. It is likely to also negatively affect educational outcomes [18] and the ability of mothers to adopt exclusive breastfeeding practices [19]. Therefore, achieving food security (FS) is necessary and urgent. It is prominently acknowledged by the United Nations through Sustainable Development Goals 2 and 3 (end hunger; ensure good health and well-being, respectively) [20]. Nevertheless, persistent food crises in the Global South has led many stakeholders, including policy makers and academics, to redefine the concept of FS. This has also resulted in various changes to the approaches used by governments and aid organizations to address FI [21].

The debate over the FS concept has evolved from the adequacy of country-level food supplies to dietary energy adequacy at the household and individual levels, plus, currently, consideration of the economic, social, nutritional, and psychological factors [22]. FAO [23] define FS as access to an adequate supply of sufficient, safe, and nutritious food that meet people’s dietary needs and food preferences for an active and healthy life. It involves three physical dimensions namely, availability, accessibility, and utilization, along with one temporal dimension, stability [24].

A critical aspect for strategies to achieve FS is the identification of food insecure households or individuals and the characterization of the nature of their insecurity through measurements. This provides the basis for monitoring the progress and impact of FS programs [25]. Thus, multiple indicators have been proposed and applied as a way to identify and monitor those most in need of food security interventions. However, these indicators are quite heterogeneous [26] and a single measure cannot capture all its dimensions [27], thus producing mixed results [28]. In Mozambique, for example, several studies, conducted in different regions and applying different proxy indicators of FS, have found different results. Proxies applied include households’ perception of FS [29], food expenditures [30, 31], number of calories obtained by each household [32], the frequency with which households have experienced different food access challenges [33], and household food insecurity access scale [34].

Furthermore, the literature on FS measurement categorize FS indicators into two groups, one group uses indirect approaches to measure adequacy of food consumption (e.g. dietary diversity and food consumption scores), while the other directly measures behaviors and lived experiences of household food security (e.g. food insecurity experience scale) [22, 35]. Hence, the application and the comparison of different FS indicators as well as the use of mixed methods are required to holistically analyze and describe FS [36]. Moreover, some indicators capture only one FS dimension, while others combine two or more of these dimensions. Nevertheless, there are indicators that are not clear about which FS dimension they measure [35] and there is still lack of consensus on the effectiveness of most of indicators used to monitor the progress of FS [22]. All indicators have strengths and weaknesses; thus, one must take into account the trade-offs while selecting an indicator. The selection of appropriate FS indicators should always take into account the dimension of food security that is intended to be measured and if the purpose is to take a more holistic view of the food security situation, the use multiple indicators is preferable over a single indicator [35, 36].

Mawoko et al. [37] and Fitawek and Hendriks [38], use multiple FS indicators to provide a holistic picture of FS status at the household-level in different regions of Mozambique, including Gurué district, and examined the effects of large-scale agricultural investments on household FS. However, these studies only used data collected at one point of time and overlooked other key drivers conditioning households’ capacity to achieve FS; thus, giving an incomplete picture of FS, which is expected to fluctuate to the better or worse than what is shown in these studies. In our study, we attempt to close this gap: (1) using data collected in the pre-harvest period and during the harvest period to capture relevant interseasonal variation of FS; (2) combining three FS indicators to capture household access to sufficient food quantity (energy), food quality (nutrient adequacy), and stability over a one year period; and (3) exploring the underlying drivers of FS at household-level in Gurué District, Central Mozambique.

### Study area

**Fig. 1:**
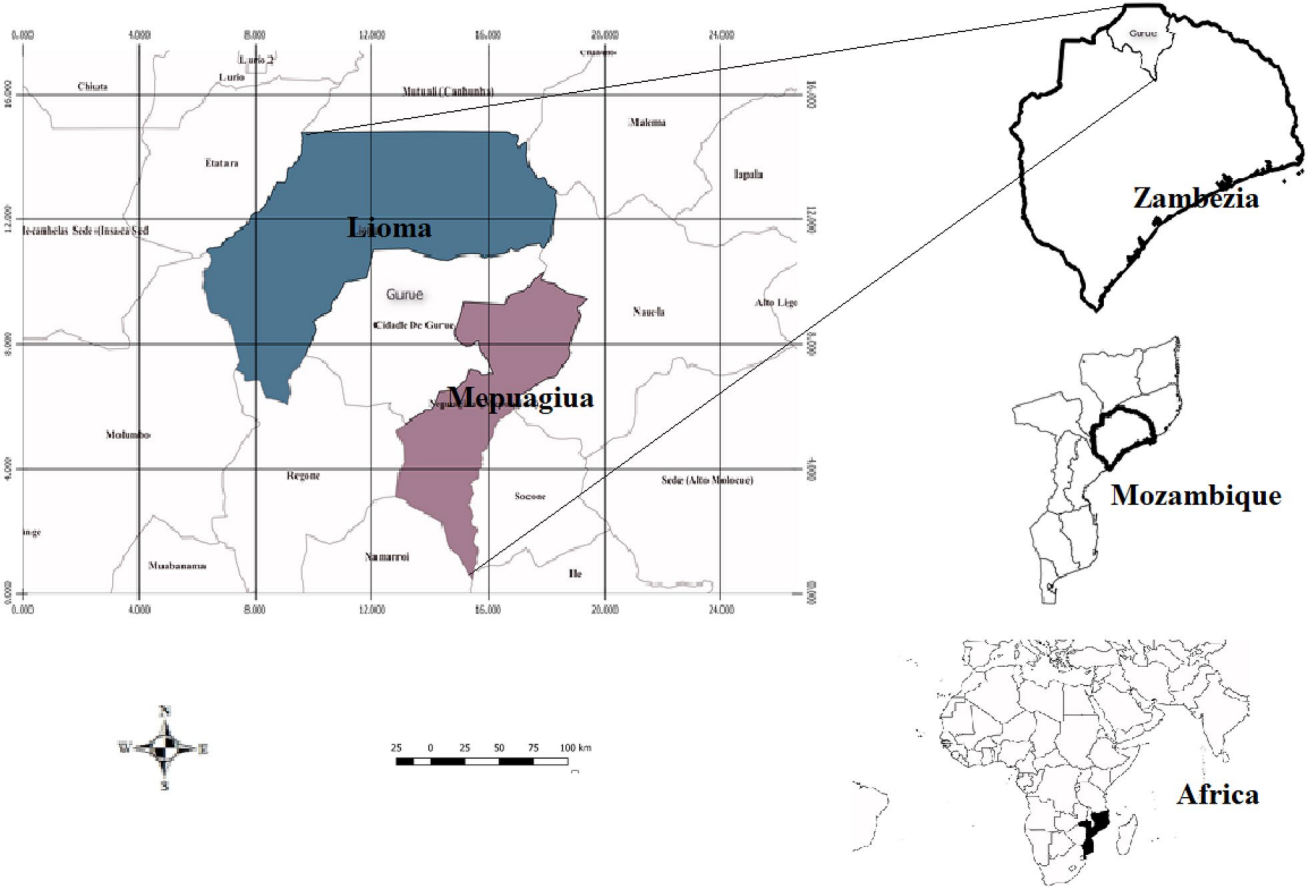
Location map for the study area

The study was conducted in two villages situated in Gurué district: Lioma (15°10′33.3"S 36°48′21.8"E) and Mepuagiua (15°46′26.4"S 37°03′38.5"E) (Fig. 1). The district is located in the north of Zambézia province, central Mozambique, bordering the Republic of Malawi. The total area of Gurué is 565,000 hectares and the population is estimated at 431,000 inhabitants, which corresponds to 7.7% of total population in Zambézia province and a population density of 76 individuals per km^2^ [39]. This area has two seasons: a rainy season, with temperatures between 30 °C and 40 °C, and a dry season with, temperatures ranging from 17 °C to 20 °C. The average annual rainfall is about 1800 mm. The main activities practiced by the population are agriculture and animal husbandry (chickens, ducks, and pigs) [39]. The agricultural activities occupy a total area of 147,760 ha, of which 10,080 ha (7%) are used primarily for large-scale commercial production; the remaining 137,680 ha (93%) is used for small-scale farming. The majority of the small-scale cultivated area is under food crop cultivation, mostly maize, cassava, and sorghum [40].

## Results

### Household characteristics

**Fig. 2:**
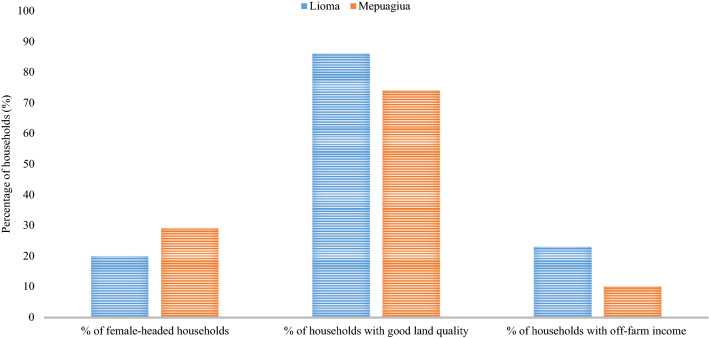
Percentage (%) of households for: gender; non-farm income; and good land quality

**Fig. 3:**
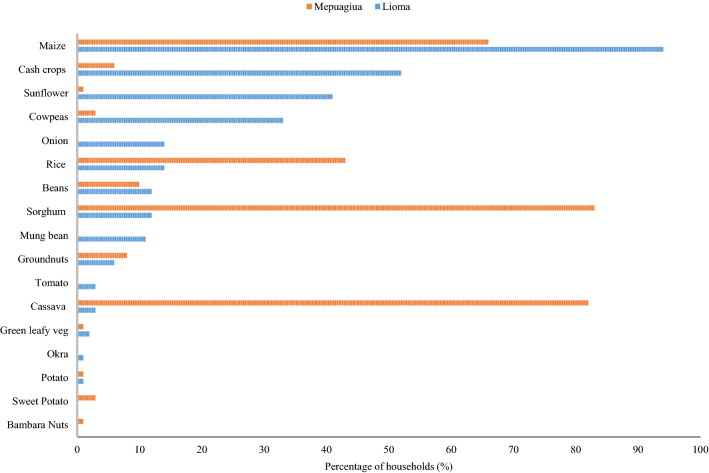
Percentage (%) of households producing specific crop in each village

The majority of households, 71% in Mepuagiua and 80% in Lioma, are headed by men. Only 10% of the households in Mepuagiua have a non-farm income source, while in Lioma, 23% of the households practice activities other than agriculture (Fig. 2). According to the results presented in Table 1, the mean age of household heads in Mepuagiua (37 years) was higher than that of Lioma (33 years) (*p* < 0.05); nevertheless, in Lioma, farmers are better educated (*p* < 0.001). The mean farm sizes are not statistically different and overall crop diversity is higher in Lioma (*p* < 0.001). Moreover, there are no statistically significant differences with regard to mean household size between both study areas. In Mepuaguia, there is more diverse staple foods production with dominance of sorghum and very little cash crop production (Fig. 3).

**Table 1:** Socioeconomic characteristics of the households in each village

Variables	Mepuagiua (*n* = 157)				Lioma (*n* = 139)				*t*-test	
	Average	Min	Max	Sd	Average	Min	Max	Sd	*t*_Critical_	*p*_(*T*_ _≤_ _*t*)_
Age of household head	36.9	18	83	14.72	33.2	18	65	10.4	1.969	0.013**
Size of the household	4.89	1	11	2.12	5.2	1	13	2.01	1.968	0.122
Farm size (ha)	1.32	0.2	5	0.85	1.39	0.2	5.5	1.01	1.969	0.565
Livestock diversity	2.73	0	4	0.56	3.06	0	6	1.14	1.971	0.671
Education of household head	4.71	0	10	2.57	5.9	0	16	2.54	1.968	< 0.001***
Crop diversity	3.27	1	9	1.31	3.87	1	9	1.68	1.969	< 0.001***

Significant levels: ***1%, **5%, *10%

### Household food security status

The household FS categories, classified via different FS indicators, are shown in Table 2. The majority of households in Mepuagiua (52%) fall into the low dietary diversity category when the HDDS is applied in the pre-harvest period, whereas in Lioma, the majority (68%) has a medium dietary diversity. Only 2% and 1% of the households fall within high dietary diversity category in Mepuagiua and Lioma, respectively, and, thus, can be assumed as food secure in pre-harvest period. However, during the harvest period, many households move into the high dietary diversity category. In Lioma, the majority (50%) present high dietary diversity and only 5% fall into low dietary diversity category. In Mepuagiua, households also increase their dietary diversity during the harvest period, with 45% having medium diet diversity and 37% having high diet diversity.

**Table 2:** Percentage of households in each food security category

FS indicators	Categories	Mepuagiua (*n* = 157)		Lioma (*n* = 139)	
		Pre-harvest (%)	Harvest (%)	Pre-harvest (%)	Harvest (%)
HDDS	High	2	37	1	50
	Medium	46	45	68	45
	Low	52	18	31	5
HFC	Acceptable	0	1	0	2
	Borderline	11	54	12	57
	Poor	89	45	88	41
MAHFP	Least FI	3	27	8	17
	Moderately FI	32	31	21	41
	Most FI	65	42	71	42

Moreover, results reveal similar trends between both study sites with regards to HFCS. The majority of the households (88% in Lioma and 89% in Mepuagiua) had a poor HFCS (HFCS ≤ 21) in the pre-harvest period. However, during the harvest period, the majority of the households are categorized as borderline. High food insecurity levels were also found with respect to MAHFP in the pre-harvest period. The majority of the households (65% in Mepuagiua and 71% in Lioma) had less than 5 MAHFP, while only 3% in Mepuagiua and 8% in Lioma were least food insecure. In the harvest period, the level of food insecure households decreased to 42% in both study sites.

### Factors affecting household food security

We used generalized ordered logit models (GOLM) with partial proportional odds (PPOM), fitted in Stata with a user-written program, *gologit2* [41] (see Additional file 1 for detailed output results). According to the PPOM results presented in Table 3, the variable Age violates the parallel line assumption in the model fitted for the dependent variables HDDS and MAHFP, meaning that their effect change across equations. The size of household and season also violates the parallel line assumption for HDDS and MAHFP, respectively. Therefore, age, size of household, and season have gamma ($\gamma$) coefficients that are statistically significant (*p* value < 0.05). To obtain the coefficient for these variables, $\gamma$ coefficients must be added to beta ($\beta$) coefficients; for example, the coefficient of season on the low and medium dietary diversities versus high dietary diversity is 4.451 (1.976 + 2.475). The two alphas ($\alpha$) represent the intercepts of each cut-off points of the logit function.

**Table 3:** Estimation results of GOLM

Variables	HDDS	MAHFP	HFCS
Beta			
Season	1.976 (0.238)^***^	1.291 (0.175)^***^	3.433 (0.310)^***^
Gender	− 0.174 (0.210)	− 0.078 (0.204)	− 0.659 (0.286)^**^
Age	− 0.026 (0.009)^***^	− 0.010 (0.008)	− 0.008 (0.010)
Geographic location	2.164 (0.433)^***^	1.078 (0.429)^**^	− 0.015 (0.598)
Size of land	− 0.114 (0.102)	0.236 (0.099)^**^	− 0.134 (0.137)
Quality of land	0.788 (0.283)^***^	1.090 (0.298)^***^	− 0.235 (0.397)
Maize	0.391 (0.253)	0.315 (0.255)	0.907 (0.368)^**^
Sorghum	0.041 (0.278)	− 0.024 (0.276)	0.314 (0.385)
Cassava	1.076 (0.314)^***^	0.689 (0.303)^**^	1.150 (0.431)^***^
Rice	0.726 (0.235)^***^	0.551 (0.228)^**^	0.327 (0.305)
Size of household	− 0.077 (0.044)^*^	− 0.045 (0.046)	− 0.079 (0.060)
Off-farm income	0.089 (0.242)	− 0.070 (0.242)	0.603 (0.324)*
Livestock diversity	0.053 (0.016)^***^	0.043 (0.014)^***^	− 0.003 (0.018)
Education	0.066 (0.038)^*^	0.043 (0.037)	0.041 (0.052)
Cash crop	0.116 (0.254)	0.349 (0.259)	0.980 (0.350)^***^
Crop diversity	0.152 (0.085)^*^	− 0.204 (0.084)^**^	0.854 (0.122)^***^
Alpha			
_cons_1	− 1.470 (0.594)^**^	− 1.8827 (0.579)^***^	− 6.590 (0.887)^***^
_cons_2	− 7.429785 (0.829)^***^	− 5.148 (0.689)^***^	− 13.082 (1.210)^***^
Gamma_2			
Age	0.022 (0.011)^**^	0.020 (0.010)^**^	
Size of household	n.a	0.129 (0.048)^***^	
Season	2.475 (0.572)^***^	n.a	

n.a, not applicable; Standard errors are presented in parenthesis; Significant levels: ***1%, **5%, *10%

All variables, except size of household, sorghum production, and off-farm income, were statistically significant at the 5% of significance level for at least one of the FS indicators used in this study (cf. Table 3). The marginal effects of each explanatory variable for FS indicators are presented in Table 4.

**Table 4:** Marginal effects of explanatory variables for FS level

Variables	HFCS categories			HDDS categories			MAHFP categories		
	Poor	Borderline	Acceptable	Low	Medium	High	Most FI	Moderately FI	Least FI
Season	− 0.570 (0.038)***	0.568 (0.038)***	0.002 (0.001)	− 0.323 (0.035)***	− 0.083 (0.044)*	0.406 (0.031)***	− 0.309 (0.039)***	0.182 (0.027)***	0.126 (0.020)***
Gender	0.126 (0.059)**	− 0.125 (0.059)**	0.000 (0.000)	0.028 (0.033)	− 0.016 (0.018)	− 0.012 (0.015)	0.019 (0.051)	− 0.012 (0.031)	− 0.008 (0.020)
Age	0.001 (0.002)	− 0.001 (0.002)	0.000 (0.000)	0.004 (0.001)***	− 0.004 (0.001)***	− 0.000 (0.000)	0.003 (0.002)	− 0.003 (0.002)*	0.001 (0.001)
Geographic location	0.003 (0.105)	− 0.003 (0.105)	0.000 (0.000)	− 0.341 (0.067)***	0.174 (0.049)***	0.167 (0.052)***	− 0.261 (0.099)***	0.154 (0.056)***	0.107 (0.046)**
Size of land	0.024 (0.024)	− 0.023 (0.024)	0.000 (0.000)	0.019 (0.017)	− 0.011 (0.010)	-0.008 (0.007)	− 0.058 (0.024)**	0.036 (0.015)**	0.022 (0.010)**
Quality of land	0.041 (0.068)	− 0.040 (0.067)	0.000 (0.000)	− 0.123 (0.042)***	0.066 (0.025)***	0.057 (0.026)**	− 0.265 (0.069)***	0.150 0.037***	0.116 (0.036)***
Maize	− 0.136 (0.047)***	0.136 (0.046)***	0.000 (0.000)	− 0.068 (0.047)	0.045 (0.034)	0.023 (0.015)	− 0.077 (0.061)	0.049 (0.040)	0.028 (0.021)
Sorghum	− 0.055 (0.068)	0.055 (0.067)	0.000 (0.000)	− 0.007 (0.045)	0.004 (0.027)	0.003 (0.018)	0.006 (0.068)	− 0.004 (0.042)	− 0.002 (0.026)
Cassava	− 0.207 (0.079)***	0.207 (0.079)***	0.001 (0.000)	− 0.170 (0.049)***	0.094 (0.032)***	0.077 (0.029)***	− 0.169 (0.073)**	0.101 (0.043)**	0.068 (0.032)**
Rice	− 0.060 (0.057)	0.059 (0.057)	0.000 (0.000)	− 0.108 (0.032)***	0.053 (0.019)***	0.055 (0.023)**	− 0.136 (0.056)**	0.079 (0.031)**	0.058 (0.026)**
Size of household	0.014 (0.011)	− 0.014 (0.011)	0.000 (0.000)	0.013 (0.007)*	− 0.008 (0.005)	− 0.005 (0.003)	0.011 (0.011)	− 0.019 (0.009)**	0.008 (0.005)
Off-farm income	− 0.117 (0.068)*	0.117 (0.068)*	0.000 (0.000)	− 0.014 (0.038)	0.008 (0.021)	0.006 (0.017)	0.017 (0.059)	− 0.011 (0.037)	− 0.007 (0.022)
Livestock diversity	0.000 (0.003)	0.000 (0.003)	0.000 (0.000)	− 0.009 (0.003)***	0.005 (0.002)***	0.004 (0.001)***	− 0.011 (0.004)***	0.007 (0.002)***	0.004 (0.001)***
Education	− 0.007 (0.009)	0.007 (0.009)	0.000 (0.000)	− 0.011 (0.006)*	0.006 (0.004)	0.004 (0.003)	− 0.011 (0.009)	0.007 (0.006)	0.004 (0.004)
Cash crops	− 0.191 (0.074)**	0.190 (0.073)**	0.001 (0.000)	− 0.019 (0.040)	0.011 (0.023)	0.008 (0.018)	− 0.086 (0.064)	0.051 (0.037)	0.035 (0.028)
Crop Diversity	− 0.150 (0.022)***	0.149 (0.022)***	0.000 (0.000)	− 0.025 (0.014)*	0.015 (0.009)*	0.010 (0.006)*	0.050 (0.021)**	− 0.031 (0.013)**	− 0.019 (0.008)**

Standard Errors are presented in parenthesis; Significant levels: ***1%, **5%, *10%

Two variables are consistently associated with all three FS indicators (*p* value < 0.05), namely, the season and the production of cassava. The three FS indicators used in this study are higher during the harvest season and for households producing cassava, which can also be seen from the negative marginal effects on the lower categories for all three indicators (cf. Table 4).

Geographic location, quality of land, livestock diversity, and rice production are statistically associated with two of the FS indicators (*p* value < 0.05). Farmers located in Lioma are more likely to have a higher HDDS and MAHFP, as shown by the negative marginal effects for the lower categories and positive marginal effects for the higher categories. The higher the quality of land, the higher the HDDS and MAHFP. The production of rice also increases HDDS and MAHFP. Households with higher livestock diversity are more likely to have higher HDDS and MAHFP. Nevertheless, crop diversity is statistically and positively related to MAHFP and HFCS.

Size of land is an important factor for ensuring adequate food provision throughout the year but does not have any influence on HDDS and HFCS. Gender is negatively associated with HFCS, which is also represented by the positive marginal effect for the lower category (0.126) and negative for the borderline (−0.125). This suggests that female-headed households are more likely to have a medium (or borderline) food consumption score compared to male-headed households. The age of the household head only affects HDDS, while the positive and negative signs of the marginal effect for the low and medium dietary diversity, respectively, indicate that the older the head of the household, the lower the dietary diversity. The production of maize and cash crops only have an effect on HFCS.

## Discussion

This study assesses the FS situation during the period immediately prior to the harvest and during the harvest period. It also deepens the analysis of the key drivers conditioning household capacity to achieve food security in Gurué district. Three FS indicators categorized into three levels were used to the capture multidimensionality and the prevalence of more nuanced patterns of FS among the households, which are neglected in those studies that sort the households into only two categories, e.g. food secure and food insecure [42]. Generally, we observe comparably low dietary diversity and predominantly poor household food consumption during the pre-harvest period and a predominance of households with medium and high dietary diversity and food consumption during the harvest season. This is in line with our expectation, as our data were collected between February and March, the period when food reserves are already scarce, and during the harvest period (May and June), when access to farm-sourced food is high [43]. As stated by [4], in Mozambique household, food security is sensitive to seasonal variations. Therefore, our results may represent both the lower and upper margins of FS in Gurué district.

It is noteworthy that gender is only associated with HFCS. According to the results presented in Table 4, female-headed households have higher food consumption scores. Due to the weighting system applied by this FS indicator, it responds more strongly to animal products, which are generally purchase in the study area. HDDS weights all food groups equally, whereas HFCS applies different weights to the different food groups [44]. Fish, which is mainly purchased in our study area, have the highest weight (4), whereas the farm-sourced vegetables and main staple, have a low weight (cf. Table 5). Thus, using HFCS may result in the highest food insecurity levels for household relying on farm-sourced food items. Although, in general, female-headed households are often behind male-headed households with regard to FS status [42, 45], behavioral differences between women and men result in differences with respect to how financial resources are spent in male and female-headed households [46]. While female decision-makers may choose to invest their financial resources in food expenditures, male decision-makers may spend their financial resources on non-food items. In line with this, studies also find that low-income women with children are motivated to improve the nutritional quality of their families and are more likely to consume a nutritious diet [47, 48].

**Table 5:** Food Groups and Item used for HFCS calculations

Food group	Food items	Weight
Main staples (MS)	Maize, Sorghum, Cassava, Rice, Potato, Sweet Potato, Bread, noodles, yam	2
Pulses (P)	Beans, Cowpeas, Mung bean, Groundnuts, Pigeon peas, soybean, Bambara Nuts	3
Vegetables (V)	Green leafy veg, Tomato, okra	1
Fruit (F)	Any fruit consumed during the seven day period	1
Meat/Fish (MF)	Beef, poultry, pork and fish	4
Dairy (D)	Milk, yogurt and other diary	4
Sugar/honey (SH)	Sugar and sugar products, honey	0.5
Fats (Fa)	Oils, fats and butter	0.5

Surprisingly, livestock diversity is statistically significant only for HDDS and MAHFP. Since HFCS weighs food groups differently, we expected households engaged in animal husbandry to have higher HFCS. However, this may imply that livestock is not used for own consumption, but rather for commercial and other purposes. Moreover, off-farm income did not have a statistically significant impact on all FS indicators (Table 4), although it is often reported as one of the most important drivers of FS [49]. Thus, the results of our study may be in line with [50], who suggested that in many regions, poor households tend to spend their income on non-food items such as clothing, household appliances, etc., and not necessarily on more nutritious foods. Notably, the production of cash crops has a positive influence on HFCS. This may suggest that cash crops are important sources of income to purchase non-farm-sourced foods, especially those with the highest weights (cf. Table 5). According to [51], households with cash crops income can purchase more appropriate and nutritious foods, thus being more likely to have improved food security. Nevertheless, the fact that the production of cash crops has a positive influence on HFCS while off-farm income does not, reinforces the importance of psychological and behavioral factors on household income expenditure and food consumption patterns [52]. Perhaps the source of income plays a role in the decision whether to invest in food or not.

The effect of age is statistically significant for HDDS, suggesting that older farmers have lower dietary diversity than younger farmers. This negative relationship between age and HDDS is also found in other studies [53, 54]. Although aged farmers are, in general, more experienced and resource endowed as compared to younger farmers [55], they may also be more likely to work fewer hours per day during the harvest season compared to younger household heads, therefore, losing their ability to diversify the diet [53].

The geographic location of the household is a statistically significant factor of food security. Households from Lioma are more food secure according to MAHFP and HDDS than those from Mepuagiua. This is partially explained by the higher crop diversity in Lioma than Mepuagiua, likely contributing to greater dietary diversity. The results indicate that crop diversity is one of the main drivers of food security, according to MAHFP and HFCS. In line with this, many existing studies demonstrate the link between crop diversity and FS [56, 57]. Another possible explanation is that the percentage of households that claim to have “good land quality” is higher in Lioma (86%) than in Mepuagiua (74%), even though the local definition of “good land quality” might differ. Studies demonstrate that this factor significantly affects agricultural productivity [58, 59] and is positively associated with MAHFP and HDDS (cf. Table 5).

The size of land only drives MAHFP. According to [60] and [61], households with larger farm size can have a comparably higher level of production diversify and produce comparably high quantities of food, therefore having a higher probability of being food secure. However, in our study area, a large farm size does not necessarily result in a higher dietary diversity. Rather, it is an essential mechanism to ensure that food is quantitatively available in most months of the year.

Our analysis further indicates that the production of the staple food crops, except sorghum, is significantly related to at least one of FS indicators. Sorghum is usually less productive and less marketable than other staples [62], thus it does not have a significant impact on FS. Rice, maize, and cassava are the most consumed crops in Mozambique [63]. Cassava ranks first in terms of average daily caloric intake by households in Mozambique (678 kilocalories per capita per day), followed by maize (478 kcal per capita per day) and rice (166 kcal per capita per day) [64]. According to our results, maize has a positive relationship only with HFCS, rice is associated to MAHFP and HDDS, while cassava is positively related to all three indicators. This might indicate that, although all food crops play an important role in ensuring at least one dimension of household food security, cassava is used as both a subsistence crop (ensuring household access to calories) and a market crop (allowing households to sell surplus and re-invest into other food groups). In addition, cassava is a drought-tolerant crop and has a low-cost vegetative propagation. Thus, it might improve households’ ability to absorb weather-induced failures in the production of other staple crops. Maize, however, is a seasonal crop, prone to weather shocks such as droughts and floods [65]. Moreover, the simple in-ground storage and perennial nature of cassava offers a flexible harvesting calendar that enables farmers to adjust harvested quantities throughout the year [66]. Likewise, local rice is resistant to floods [67] and usually goes through manual processing [68], thus it remains stored for a slightly longer period, which increases its availability to the households over the year but is less likely to simultaneously satisfy the households needs and produce marketable surplus. Although the staple food crops are mostly grown for household consumption [40], maize and cassava are positively related to HFCS. This is because they are important sources of marketable surpluses that can improve income and the ability to re-invest into other food groups [69]. In fact, more than 50% of households sell at least a part of the maize they produce. In general, however, in our study region, farmers consume approximately 60% and sell 40% of their agricultural products to cover non-food household expenses [62].

The percentage of food secure households differs depending on the individual indicator applied (Table 4). This underlines that each indicator reflects the eventual output of the different FS dimensions (especially availability, access, and stability) [27]. Maxwell et al*.* [28] likewise find that different food security measures can produce divergent results. Although, HFCS and HDDS share a common emphasis on dietary diversity as a proxy for household food access and are both correlated with total per capita food and non-food expenditures, they are not interchangeable [44]. Unlike HDDS, the weights applied by HFCS do not reflect per capita calories intake but rather reflect the quality of the diet [70], since including animal source foods, fruits, vegetables, and dairy products in the diets increases the intake of essential micronutrients [71]. This is an important aspect as programs to overcome FI may follow different agricultural production strategies, depending on the individual food security indicator applied or weighted most. Policies aiming to raise, for example, diet quality would promote more market-oriented agriculture with the aim of improving HFCS, while an increase of calories intake may be achieved when production for own consumption is promoted. Hence, our findings support the idea of a fair balance between the production of cash crops and crops for own consumption [72]. MAHFP has a recall period of 12 months and, thus, it is a useful tool to capture food stability [73], as it shows households’ ability to address vulnerability over the year.

Food utilization is an essential dimension of FS that encompasses the nutritional quality of food within households and the bioavailability of nutrients in those foods. The traditional proxy measure of food utilization is the use anthropometric measurements, e.g. nutritional status, to understand whether food is allocated equally to all individual household members [35]. Nevertheless, our study is conducted at household level, thus unable to capture this dimension. Therefore, it is essential for future research to combine the self-reported consumption patterns with some FS outcomes, i.e., anthropometric measures, to understand whether food is allocated equally to all individual household members. This generates more complete information for policy makers and development agencies. The short recall period applied by HDDS and HFCS (7 days) represents another limitation of this study, though these indicator are very useful and sensitive enough to show dietary diversity at the household level [27].

## Conclusions

In this study, we investigate the FS situation and its drivers at the household level in Gurué District, Zambézia province, central Mozambique by applying different indicators (HDDS, HFCS, MAHFP). In general, we find very critical levels of food insecurity among households during the pre-harvest season and relatively high levels of food security during the harvest season. However, varying outcomes are found depending on the indicator used to assess FS. Thus, the decision whether farmers should rely on staple foods production for increasing their FS status, or specialize in cash crop production to generate income and buy food items depends on the indicator used to assess FS. For instance, MAHFP and HDDS are more related to the consumption of farm-sourced food, while HFCS responds more strongly to purchased food. As such, combining different food security indicators is an important strategy to holistically assess the food security status of the local population. In our analysis, we find that gender and age of the household head, geographic location, size and quality of land, staples production (especially cassava), livestock, crop diversity and cash crops are important drivers of at least one of FS indicator. Thus, one central recommendation derived from our results is that relevant stakeholders can improve FS by promoting a balance between market-oriented agriculture and subsistence production, as well as by supporting farmers whose land is of low quality, e.g., by teaching composting techniques to enhance agricultural productivity. To reduce the seasonality of food security, policy makers and relevant agencies must direct their efforts at increasing access to irrigation technologies to offset the drought-related shocks that occur during the dry season.

## Methods

### Study and sample design

In this study, the purpose is to assess the prevalence and the factors associated with FS during the period immediately prior to the harvest and during the harvest season, thus, a two-wave panel study design is used to collect both qualitative and quantitative data. Study participants were randomly selected from lists provided by the local administrative office and training records provided by local extension services. Before selecting the households, we combined the two lists and removed duplicates. The sample size was determined using the equation by [74]:

$$n = \frac{{Z^{2} *p*q}}{{d^{2} }},$$

where $n$ = sample size; $Z$ = abscissa of the standard normal curve (*Z* = 1.96 for 95% confidence); $p$ = proportion of agriculture-based households (0.9 for Lioma e 0.89 for Mepuagiua); $d$ = error set at 0.05; and $q = 1 - p$. The resulting sample sizes are demonstrated in Eq. 2.

1 $$n_{L} = \frac{{1.96^{2} *0.9*0.1}}{{0.05^{2} }} = 138$$

2 $$n_{M} = \frac{{1.96^{2} *0.89*0.11}}{{0.05^{2} }} = 150,$$

where $n_{L}$ = total sample size in Lioma and $n_{M}$ = total sample size in Mepuagiua. Nevertheless, one and seven additional households were included in the samples in Lioma and Mepuagiua, respectively. Thus, the survey covered 296 households (*n* = 157 in Mepuagiua and *n* = 139 in Lioma).

### Data collection

We use panel data that was collected in two waves through a semi-structured household survey questionnaire. The first wave took place in February and March 2020 and the second wave in May and June 2021. Priority was given to household heads as responding individuals. Nonetheless, the head of the household was not available in 58 (32 in Mepiagiua and 26 in Lioma) and 47 (24 in Mepiagiua and 23 in Lioma) of the selected households in the first and second waves, respectively. Thus, in these households, we interviewed any available adult household member (> 18 year old). All respondents agreed to participate in the survey and signed a consent form, translated into the Portuguese language. Through the survey, we collected data on household socio-economic characteristics and demographics, crop production and food consumption patterns focusing on specific FS indicators (HDDS, HFCS and MAHFP). To access the full dataset, see Additional file 2.

#### Indicators of food security

In this study, we select indicators that capture household access to sufficient food quantity and quality, namely, Household Dietary Diversity Score (HDDS), Household Food Consumption Score (HFCS) [35, 75]. The stability of FS over a period of 1 year is assessed using Months of Adequate Household Food Provisioning (MAHFP) [73]. These indicators are detailed in the following sub-sections.

*Household dietary diversity score (HDDS)* The HDDS is a proxy measure of household nutrient adequacy [76]. It lists the number of different food groups consumed over a given reference period [77], thus capturing dietary quality within a household [76]. Households were asked to report the foods (meals and snacks) that they ate or drank during the 7 days prior to the survey to capture variability in intake. Subsequently, the food items were grouped into 12 food groups, as defined by Kennedy et al*.* [78], to calculate the HDDS for each household: Cereals, fish and seafood, root and tubers, pulses/legumes/nuts, vegetables, milk and milk products, fruits, oil/fats, meat/poultry, sugar/honey, eggs and miscellaneous. Values for the food groups were either 1 (consumed) or 0 (not consumed). Thus, HDDS is the total number of food groups consumed by household members. Households were classified as having low (HDDS ≤ 3), medium (HDDS = 4–6), or high dietary diversity (HDDS = 7–12).

*Household food consumption score (HFCS)* The HFCS is a widely applied food security indicator that is calculated using the frequency of consumption of different food groups by a household during a 7 days period [79]. Household members were asked the question: “How many days in the past 7 days prior to the survey did the household eat each of the food items presented in Table 1?” Following this, the data were grouped into three food consumption groups, subsequently combined into a composite score using standardized weights (Table 5). We created the food consumption groups using standard thresholds, classifying a household’s food consumption as being poor (HFCS ≤ 21), borderline (HFCS = 21.5–35), or acceptable (HFCS > 35) [79]. HFCS is calculated by Eq. 3 (respective abbreviations are displayed in the subsequent Table 5).

3 $${\text{HFCS }} = \left( {{\text{MS}}*2} \right) + \left( {P*3} \right) + V +F + \left( {{\text{MF}}*4} \right) +\left( {D*4} \right) + \left( {{\text{Fa}}*0.5} \right) + \left( {{\text{SH}}*0.5} \right)$$

*Months of adequate household food provisioning (MAHFP)* The MAHFP is an indicator that captures the consistency of food availability throughout a 12 months period [73]. Respondents were asked to identify in which of the last 12 months they had access to sufficient food to meet their household needs, jointly defined with villagers as the months they could have at least three meals a day. Values for each month were either 1 (yes) or 0 (no). Therefore MAHFP was the total number of months all household members could have three or more meals a day. Households were classified as most food insecure (MAHFP ≤ 5), moderately food insecure (MAHFP = 6–9), or least food insecure (MAHFP = 10–12).

### Data analysis

#### Descriptive statistics

The first step of data analysis was to determine the profile of farming activities and the socio-economic characteristics of the study area. We computed basic descriptive statistics to provide summary statistics of the data. Frequency distributions and percentage were used for categorical variables. Mean, minimum, maximum, and standard deviation were used for continuous variables. Due to the differences in sample sizes, Welch’s *t* test was used to compare the means of the two study sites [80].

#### Generalized ordered logit model (GOLM)

The food security indicators used is this study were categorized into three levels (cf. Table 2), coded as 0 = lower, 1 = medium and 2 = higher level. Thus, these indicators represent the level of FS in an ordinal scale with the level *j* = 0 being the minimum value of the indicators (low = 0 for HDDS; poor = 0 for FCS; most FI = 0 for MAHFCS). For such ordinal dependent variables, ordered logit model is generally suggested [81]. However, the ordered logit model must meet the proportional odds assumption (also known as parallel line assumption), such that the coefficients of explanatory variables on different levels of the dependent variable are the same across different cut points [82], but this is usually not the case [41]. In this study, we initially applied the ordered probability models, however, the Brant test [83] suggested that the parallel line assumption was violated, which means that a subset of variables has a varying coefficient. Thus, to identify the factors that affect the different food security indicators, we applied the generalized ordered logit models with partial proportional odds model (PPOM) for each FS indicator (MAHFP, HFCS, and HDDS) using the program *gologit2* in Stata [41]. This model is already applied in studies assessing factors associated to food security, e.g., Akbar et al*.* [84] and Ayele et al*.* [85]. PPOM relaxes the restriction of parallel line assumption, allowing one or more coefficients to differ across equations while others can be the same for all equations. The general model is presented in Eq. 4.

4 $$P\left( {Y_{i} > j} \right) = \frac{{{\text{exp}}\left( {\alpha_{j} + X_{i1} \beta_{j1} + X_{i2} \beta_{2} } \right)}}{{1 + \left[ {\exp \left( {\alpha_{j} + X_{i1} \beta_{j1} + X_{i2} \beta_{2} } \right)} \right]}} , j = 1,2, \ldots , m - 1$$

where $Y_{i}$ is the recorded FS category for household $i$. $P\left( {Y_{i} > j} \right)$ is the probability of a household $i$ be in a given FS category. $j$ is the number of categories or cut points. $\alpha_{j}$ is the regression intercept of each category. $m$ is the number of categories of the FS indicators (c.f Table 2). $X_{i1}$ is the vector of explanatory variables that violate the constraint of parallel line assumption. $\beta_{j1}$ is a vector of regression coefficients that varies across the category. $X_{i2}$ is the vector of the rest of the explanatory variables with a vector of regression coefficients $\beta_{2}$.

As we intended to have more parsimonious layout to easily pinpoint the variables violating the assumptions, we used a gamma parameterization (see Additional file 1 for detailed output results), an equivalent form of partial proportional odds model proposed by Peterson et al*.* [86]. The model is presented in Eq. 5.

5 $$P\left( {Y_{i} > j} \right) = \frac{{{\text{exp}}\left( {\alpha_{j} + X_{i} \beta + T_{i} \gamma_{i} } \right)}}{{1 + \left[ {\exp \left( {\alpha_{j} + X_{i} \beta + T_{i} \gamma_{i} } \right)} \right]}} , j = 1,2, \ldots , m - 1$$

where $T_{i}$ is the vector of explanatory variables that violate the assumption of proportional odds. $\beta$ is the effect of variables that have the same coefficients for all possible pairs of FS categories, while $\gamma_{i}$ is the differential effect of the variables on each pair of FS categories and indicate the extent to which the parallel regression assumption is violated by the variable. Each explanatory variable violating the parallel line assumption has one $\beta$ coefficient and $m - 1$ $\gamma$ coefficients [41]. Thus, if $\gamma_{i} = 0$ the model would reduce to the ordered logit model [87]. The explanatory variables used in PPOM and the expected signs are summarized in Table 6. These variables were selected due to their potential explanatory power for food security, based on prior identified publications.

**Table 6:** Explanatory variables used in the regression analysis

Variable	Description	Expected signs
Age	Age of household head in years	+
Education	Numbers of schooling years of household head	+
Gender	Gender of household head (1 = male, 0 = female)	+
Household size	Number of household members	−
Extra income	Off-farm/non-farm income (1 if yes, 0 if no)	+
Livestock diversity	Number of animal species raised by the household	+
Location	Household location (Lioma = 1; Mepuagiua = 0)	±
Farm size	Farm size in hectares	+
Land quality	Perception of having good land fertility (good = 1; bad = 0)	+
Crop diversity	Number of crop species produced by the household	+
Maize	Maize production (1 if yes, 0 if no)	+
Sorghum	Sorghum production (1 if yes, 0 if no)	+
Rice	Rice production (1 if yes, 0 if no)	+
Cassava	Cassava production (1 if yes, 0 if no)	+
Cash crops^a^	Soybean and/or Tobacco production (1 if yes, 0 if no)	+
Season	Period of data collection (1 = harvest season, 0 = pre-harvest season)	+

^a^Soybean and tobacco are the major cash crops in the study area

The age of the household head is a continuous variable that is used as a proxy for farming experience [88, 89]. Older farmers are assumed to be more experienced and resource endowed as compared to younger farmers [55]. Thus, we expect in our study that food security increases with age. Education of the household head is expected to have a positive impact on FS, as educated farmers are better able to obtain information on improved agricultural techniques and new economic opportunities, thereby increasing their productivity [90, 91]. Moreover, it is already demonstrated that education positively affects FS [92–94]. The knowledge gap and gender differences in access, control, and use of assets is a major concern of gender studies in agriculture [95, 96]. Therefore, we expect differences between male and female-headed households with regards to food security status. This is a dummy variable equaling 1 if the household head is male and 0 if the household head is female. The size of the household is the total number of people that depend and live in a household. Although some literature suggests that lager households may reflect household labor available for agricultural activities [51], we expect households with a larger number of people to be less food secure, since larger households have a higher burden to feed [61, 88]. Geographic location is a dummy variable that is equal to 1 for a household located in Lioma and 0 for households in Mepuagiua. Due to differences in some socio-economic characteristics, we expect differences in food security status between the two villages.

Households with off-farm and cash crop income can purchase more appropriate and nutritious foods, thus improving food security [97]. As such, we expect these households to be more likely to be food secure than those without non-farm and cash crops income. These are dummy variables that equal 1 for households with non-farm and cash crops (soybean and/or tobacco) income and 0 otherwise. Livestock and crop diversity represent the number of animal and crop species raised and produced by a household, respectively. Livestock ownership represents an additional source of subsistence, income, and nutritional requirements [88]. Furthermore, several studies demonstrate the link between crop diversity and FS [54, 56, 57]. Therefore, we expect these variables to have a positive relationship with FS.

Farm size is a continuous variable and measured in hectares. In this study, we expect households with larger farm sizes to have higher probability of being food secure, since they can diversify production and produce more quantities [60, 61]. Moreover, we expect the production of staple foods to be significantly related to FS. Staple foods represent the foundation for food security and an adequate diet [98]. They are sources of marketable surpluses that can improve income and the ability to purchase foods other than those produced within the household [69]. Rice, sorghum, maize and cassava were selected in this study, as they are the most produced and consumed staple crops in the study region. Each staple crop represented a dummy variable that equals 1 if the crop was produced by the household and 0 otherwise. Quality of land is a dummy and represent the perception of having good land fertility. It is equal to 1 if the household assumes it has good land quality and is expected to be positively related to food security. The definition of “good” is based on the individual farmers’ assessment of their own land. Season is an important factor for food security in Mozambique [4], thus, we expect an increase of food security during the harvest season.

## Supplementary Information

**Additional file 1:** Results output. Detailed results output of the partial proportional odds model.

**Additional file 2:** Data set. Data set used in this study.

## Data Availability

All data generated and analyzed during this study are included in Additional file 2.

## References

[1] FAO, ECA, AUC. Africa regional overview of food security and nutrition 2019. Accra, Ghana: FAO; 2020.

[2] WFP. WFP Mozambique: country Brief September 2019. WFP Mozambique Country Brief 2019; 2019.

[3] USAID, WFP. USAID Mozambique: World Food Program Fact Sheet, February 2020. USAID; 2020.

[4] Selvester K, Fidalgo L, Ballard T, Kennedy G, Dop M, Mistura L, et al. Report on use of the household food insecurity access scale and household dietary diversity score in two survey rounds in Manica and Sofala Provinces, Mozambique. FAO Project. 2008; 1–23.

[5] Rose ES, Blevins M, González-Calvo L, Ndatimana E, Green AF, Lopez M (2015). Determinants of undernutrition among children aged 6 to 59 months in rural Zambézia Province, Mozambique: results of two population-based serial cross-sectional surveys. BMC Nutr.

[6] UNICEF. Nutrition situation in Mozambique 2016 https://www.unicef.org/mozambique/en/nutrition. Accessed 19 May 2020.

[7] Artur L, Hilhorst D (2012). Everyday realities of climate change adaptation in Mozambique. Glob Environ Chang.

[8] Adhikari U, Nejadhashemi AP, Woznicki SA (2015). Climate change and eastern Africa: a review of impact on major crops. Food Energy Security.

[9] USAID. Food Assistance Fact Sheet – Mozambique-June 12, 2019. USAID; 2019.

[10] Bey A, Jetimane J, Lisboa SN, Ribeiro N, Sitoe A, Meyfroidt P (2020). Mapping smallholder and large-scale cropland dynamics with a flexible classification system and pixel-based composites in an emerging frontier of Mozambique. Remote Sens Environ.

[11] Barrett CB. Actions now can curb food systems fallout from COVID-19. Nature Food. 2020. 10.1038/s43016-020-0085-y 37128084

[12] García Cruz LM, González Azpeitia G, Reyes Súarez D, Santana Rodríguez A, Loro Ferrer JF, Serra-Majem L (2017). Factors associated with stunting among children aged 0 to 59 months from the central region of Mozambique. Nutrients.

[13] Hadley C, Stevenson EGJ, Tadesse Y, Belachew T (2012). Rapidly rising food prices and the experience of food insecurity in urban Ethiopia: impacts on health and well-being. Soc Sci Med.

[14] Maitra C, Sethi V, Unisa S, Shankar S (2019). Household food insecurity and maternal and child nutritional status: evidence from Maharashtra. Rev Income Wealth.

[15] Diamond KK, Stebleton MJ, delMas RC. Exploring the relationship between food insecurity and mental health in an undergraduate student population. J Student Affairs Res Pract. 2019; 1–15.

[16] Whittle HJ, Sheira LA, Wolfe WR, Frongillo EA, Palar K, Merenstein D (2019). Food insecurity is associated with anxiety, stress, and symptoms of posttraumatic stress disorder in a cohort of women with or at risk of HIV in the United States. J Nutr.

[17] Masa R, Graham L, Khan Z, Chowa G, Patel L (2019). Food insecurity, sexual risk taking, and sexual victimization in Ghanaian adolescents and young South African adults. Int J Public Health.

[18] Cady CL (2014). Food insecurity as a student issue. J College Char.

[19] Maitra C (2018). A review of studies examining the link between food insecurity and malnutritio.

[20] UN. The 17 Sustainable development goals: United Nations; 2015 https://sdgs.un.org/goals. Accessed 09 Sep 2020.

[21] Haysom G, Tawodzera G (2018). “Measurement drives diagnosis and response”: gaps in transferring food security assessment to the urban scale. Food Policy.

[22] Cafiero C, Melgar-Quiñonez HR, Ballard TJ, Kepple AW (2014). Validity and reliability of food security measures. Ann N Y Acad Sci.

[23] FAO. World food summit plan of action. Rome: FAO; 1996.

[24] Gross R, Schoeneberger H, Pfeifer H, Preuss H-J. The four dimensions of food and nutrition security: definitions and concepts. 2000. Contract No.: 20.

[25] Hoddinott J. Choosing outcome indicators of household food security: Citeseer; 1999.

[26] Santeramo FG (2015). On the composite indicators for food security: decisions matter!. Food Rev Intl.

[27] Hendriks SL, van der Merwe C, Ngidi MS, Manyamba C, Mbele M, McIntyre AM (2016). What are we measuring? Comparison of household food security indicators in the Eastern Cape Province, South Africa. Ecol Food Nutr.

[28] Maxwell D, Vaitla B, Coates J (2014). How do indicators of household food insecurity measure up? An empirical comparison from Ethiopia. Food Policy.

[29] Pitoro R, Chagomoka T (2017). Food security dynamics and its drivers in rural Mozambique. Int J Sci.

[30] Garrett JL, Ruel MT (1999). Are determinants of rural and urban food security and nutritional status different? Some Insights from Mozambique. World Dev.

[31] Nyyssölä M, Pirttilä J, Sandström S. Helping poor farmers to help themselves: Evidence from a group-based aid project in Mozambique. WIDER Working Paper; 2012. Report No.: 9292305522.

[32] Mabiso A, Cunguara B, Benfica R (2014). Food (In)security and its drivers: insights from trends and opportunities in rural Mozambique. Food Security.

[33] McCordic C, Abrahamo E (2019). Family structure and severe food insecurity in Maputo and Matola, Mozambique. Sustainability.

[34] Riley L, Caesar M (2018). Urban household food security in China and Mozambique: a gender-based comparative approach. Dev Pract.

[35] Jones AD, Ngure FM, Pelto G, Young SL (2013). What are we assessing when we measure food security? A compendium and review of current metrics. Adv Nutr.

[36] Carletto C, Zezza A, Banerjee R (2013). Towards better measurement of household food security: harmonizing indicators and the role of household surveys. Glob Food Sec.

[37] Mawoko Z, Hendriks S, Reys A. The influence of large-scale agricultural investments on household food security in the Gurue and Monapo districts of Mozambique; 2018.

[38] Fitawek W, Hendriks SL (2021). Evaluating the impact of large-scale agricultural investments on household food security using an endogenous switching regression model. Land.

[39] INE. Folheto estatistico distrital gurue 2018. Quelimane: Instituto Nacional de Estatística; 2018.

[40] Soares MG (2017). Relação entre as mudanças de uso e cobertura de terra e as queimadas em florestas de Miombo, Gurué.

[41] Williams R (2006). Generalized ordered logit/partial proportional odds models for ordinal dependent variables. Stand Genomic Sci.

[42] Gebre GG, Isoda H, Amekawa Y, Rahut DB, Nomura H, Watanabe T (2021). What explains gender gaps in household food security? Evidence from maize farm households in Southern Ethiopia. Soc Indic Res.

[43] Matavel C, Hoffmann H, Rybak C, Sieber S (2020). Can subsistence farming help to achieve household food security? Evidence from Gurue, Central Mozambique.

[44] Kennedy G, Berardo A, Papavero C, Horjus P, Ballard T, Dop M (2010). Proxy measures of household food consumption for food security assessment and surveillance: comparison of the household dietary diversity and food consumption scores. Public Health Nutr.

[45] Broussard NH (2019). What explains gender differences in food insecurity?. Food Policy.

[46] Carranza M, Niles MT (2019). Smallholder farmers spend credit primarily on food: gender differences and food security implications in a changing climate. Front Sustain Food Syst..

[47] Evans A, Chow S, Jennings R, Dave J, Scoblick K, Sterba KR (2011). Traditional foods and practices of spanish-speaking Latina mothers influence the home food environment: implications for future interventions. J Am Diet Assoc.

[48] Dubowitz T, Acevedo-Garcia D, Salkeld J, Cristina Lindsay A, Subramanian SV, Peterson KE (2007). Lifecourse, immigrant status and acculturation in food purchasing and preparation among low-income mothers. Public Health Nutr.

[49] Frelat R, Lopez-Ridaura S, Giller KE, Herrero M, Douxchamps S, Djurfeldt AA (2016). Drivers of household food availability in sub-Saharan Africa based on big data from small farms. Proc Natl Acad Sci.

[50] Banerjee AV, Duflo E. Poor economics: a radical rethinking of the way to fight global poverty: public affairs; 2011.

[51] Mango N, Zamasiya B, Makate C, Nyikahadzoi K, Siziba S (2014). Factors influencing household food security among smallholder farmers in the Mudzi district of Zimbabwe. Dev South Afr.

[52] Silva A, Caro JC, Magaña-Lemus D (2016). Household food security: perceptions, behavior and nutritional quality of food purchases. J Econ Psychol.

[53] Huluka AT, Wondimagegnhu BA (2019). Determinants of household dietary diversity in the Yayo biosphere reserve of Ethiopia: an empirical analysis using sustainable livelihood framework. Cogent Food Agric.

[54] Jones AD, Shrinivas A, Bezner-Kerr R (2014). Farm production diversity is associated with greater household dietary diversity in Malawi: findings from nationally representative data. Food Policy.

[55] Etwire PM, Martey E, Dogbe W. Technical efficiency of soybean farms and its determinants in Saboba and Chereponi districts of northern Ghana: a stochastic frontier approach. 2013.

[56] Rajendran S, Afari-Sefa V, Shee A, Bocher T, Bekunda M, Lukumay PJ (2017). Does crop diversity contribute to dietary diversity? Evidence from integration of vegetables into maize-based farming systems. Agric Food Security.

[57] Snapp SS, Fisher M (2015). “Filling the maize basket” supports crop diversity and quality of household diet in Malawi. Food Security.

[58] Wiebe KD. Linking land quality, agricultural productivity, and food security. USDA-ERS Agricultural Economic Report. 2003(823).

[59] Sanchez PA (2002). Soil fertility and hunger in Africa. Science.

[60] Van der Veen A, Gebrehiwot T (2011). Effect of policy interventions on food security in Tigray, Northern Ethiopia. Ecol Soc.

[61] Aidoo R, Mensah JO, Tuffour T. Determinants of household food security in the Sekyere-Afram plains district of Ghana. Eur Sci J. 2013;9(21).

[62] Khatiwada L, Mussagy IH, dos Anjos LA, Siniquinha A, Biedronski J (2021). Zambézia market analysis report.

[63] Popat M, Tostão E, Fontes F, Vilanculos OC. Monitoring price incentives for rice in Mozambique. 2017.

[64] NET F. Mozambique Staple Food Market Fundamentals, September 2018. USAID 2019.

[65] Salazar C, Ayalew H, Fisker P (2019). Weather shocks and spatial market efficiency: evidence from Mozambique. J Dev Studies.

[66] Haggblade S, Andersson Djurfeldt A, Banda Nyirenda D, Bergman Lodin J, Brimer L, Chiona M (2012). Cassava commercialization in Southeastern Africa. J Agribusiness Dev Emerg Econ.

[67] Salazar-Espinoza C, Jones S, Tarp F (2015). Weather shocks and cropland decisions in rural Mozambique. Food Policy.

[68] Vellema S, Beekman W. Rice farmers, local markets and rice trade: a case study of local market dynamics in Mozambique and its implications for a cooperative model. Wageningen International/DGIS; 2011.

[69] Mango N, Makate C, Mapemba L, Sopo M (2018). The role of crop diversification in improving household food security in central Malawi. Agric Food Secur.

[70] Wiesmann D, Bassett L, Benson T, Hoddinott J. Validation of the world food programme s food consumption score and alternative indicators of household food security: Intl Food Policy Res Inst; 2009.

[71] Wiesmann D. A global hunger index: measurement concept, ranking of countries, and trends: Intl Food Policy Res Inst; 2006.

[72] Ntakyo PR, van den Berg M (2019). Effect of market production on rural household food consumption: evidence from Uganda. Food Security.

[73] Bilinsky P, Swindale A. Months of adequate household food provisioning (MAHFP) for measurement of household food access: indicator guide: food and Nutritional Technical Assistance Project, Academy for Educational; 2007.

[74] Cochran WG (1963). Sampling technique.

[75] Leroy JL, Ruel M, Frongillo EA, Harris J, Ballard TJ (2015). Measuring the food access dimension of food security: a critical review and mapping of indicators. Food Nutr Bull.

[76] Swindale A, Bilinsky P (2006). Household dietary diversity score (HDDS) for measurement of household food access: indicator guide.

[77] Hoddinott J, Yohannes Y. Dietary diversity as a food security indicator. 2002.

[78] Kennedy G, Ballard T, Dop M (2010). Guidelines for measuring household and individual dietary diversity.

[79] WFP. Food consumption analysis: Calculation and use of the food consumption score in food security analysis. World Food Programme; 2008.

[80] Sakai T, editor Two sample t-tests for ir evaluation: Student or welch? Proceedings of the 39th International ACM SIGIR conference on Research and Development in Information Retrieval; 2016.

[81] Walker SH, Duncan DB (1967). Estimation of the probability of an event as a function of several independent variables. Biometrika.

[82] Wang J, Wang Y, Peng Y, Lu JJ (2021). Examining partial proportional odds model in analyzing severity of high-speed railway accident. Smart Resilient Transport.

[83] Brant R (1990). Assessing proportionality in the proportional odds model for ordinal logistic regression. Biometrics.

[84] Akbar M, Niaz R, Amjad M (2020). Determinants of households’ food insecurity with severity dimensions in Pakistan: varying estimates using partial proportional odds model. Health Soc Care Community.

[85] Ayele AW, Kassa M, Fentahun Y, Edmealem H (2020). Prevalence and associated factors for rural households food insecurity in selected districts of east Gojjam zone, northern Ethiopia: cross-sectional study. BMC Public Health.

[86] Peterson B, Harrell FE (1990). Partial proportional odds models for ordinal response variables. J Roy Stat Soc Ser C (Appl Stat).

[87] Michalaki P, Quddus MA, Pitfield D, Huetson A (2015). Exploring the factors affecting motorway accident severity in England using the generalised ordered logistic regression model. J Safety Res.

[88] Bogale A, Shimelis A (2009). Household level determinants of food insecurity in rural areas of Dire Dawa, Eastern Ethiopia. Afr J Food Agric Nutr Dev.

[89] Tauer LW, editor. Farmer productivity by age over eight US census years. International Farm Management Association Conference; 2017.

[90] Asfaw A, Admassie A (2004). The role of education on the adoption of chemical fertiliser under different socioeconomic environments in Ethiopia. Agric Econ.

[91] Weir S, Knight J (2004). Externality effects of education: dynamics of the adoption and diffusion of an innovation in rural Ethiopia. Econ Dev Cult Change.

[92] Fisher M, Lewin PA (2013). Household, community, and policy determinants of food insecurity in rural Malawi. Dev South Afr.

[93] Idrisa Y, Gwary M, Shehu H (2008). Analysis of food security status among farming households in Jere Local Government of Borno State, Nigeria. Agro-Science..

[94] Makombe T, Lewin P, Fisher M. The determinants of food insecurity in rural Malawi: implications for agricultural policy. International Food Policy Research Institute (IFPRI); 2010.

[95] Croppenstedt A, Goldstein M, Rosas N (2013). Gender and agriculture: inefficiencies, segregation, and low productivity traps. World Bank Res Observer.

[96] Kilic T, Winters P, Carletto C (2015). Gender and agriculture in sub-Saharan Africa: introduction to the special issue. Agric Econ.

[97] Kuma T, Dereje M, Hirvonen K, Minten B (2019). Cash crops and food security: evidence from Ethiopian smallholder coffee producers. J Dev Studies.

[98] FAO. The Special Programme for Food Security. FAO; 1996.

